# In Vivo Evolution of a Catalytic RNA Couples Trans-Splicing to Translation

**DOI:** 10.1371/journal.pone.0086473

**Published:** 2014-01-23

**Authors:** Karen E. Olson, Gregory F. Dolan, Ulrich F. Müller

**Affiliations:** Department of Chemistry and Biochemistry, University of California San Diego, La Jolla, California, United States of America; Max-Planck-Institute for Terrestrial Microbiology, Germany

## Abstract

How does a non-coding RNA evolve in cells? To address this question experimentally we evolved a trans-splicing variant of the group I intron ribozyme from *Tetrahymena* over 21 cycles of evolution in *E.coli* cells. Sequence variation was introduced during the evolution by mutagenic and recombinative PCR, and increasingly active ribozymes were selected by their repair of an mRNA mediating antibiotic resistance. The most efficient ribozyme contained four clustered mutations that were necessary and sufficient for maximum activity in cells. Surprisingly, these mutations did not increase the trans-splicing activity of the ribozyme. Instead, they appear to have recruited a cellular protein, the transcription termination factor Rho, and facilitated more efficient translation of the ribozyme’s trans-splicing product. In addition, these mutations affected the expression of several other, unrelated genes. These results suggest that during RNA evolution in cells, four mutations can be sufficient to evolve new protein interactions, and four mutations in an RNA molecule can generate a large effect on gene regulation in the cell.

## Introduction

To answer the question how a specific macromolecule evolved requires understanding the circumstances in its evolutionary history that led to its current role [1]. However, the information of the evolutionary context and of evolutionary intermediates is usually lost to history. Instead, the biological evolution of macromolecules can be recapitulated using experimental evolution systems. Our focus is on the evolution of catalytic RNAs (ribozymes), which was studied previously by in vitro evolution experiments [2], [3], [4], [5], [6], [7], [8], [9]. We set out to study the evolution of RNAs in cells [10] because the biological evolution of RNAs may be strongly affected by their interaction with the cellular environment.

**Figure 1:**
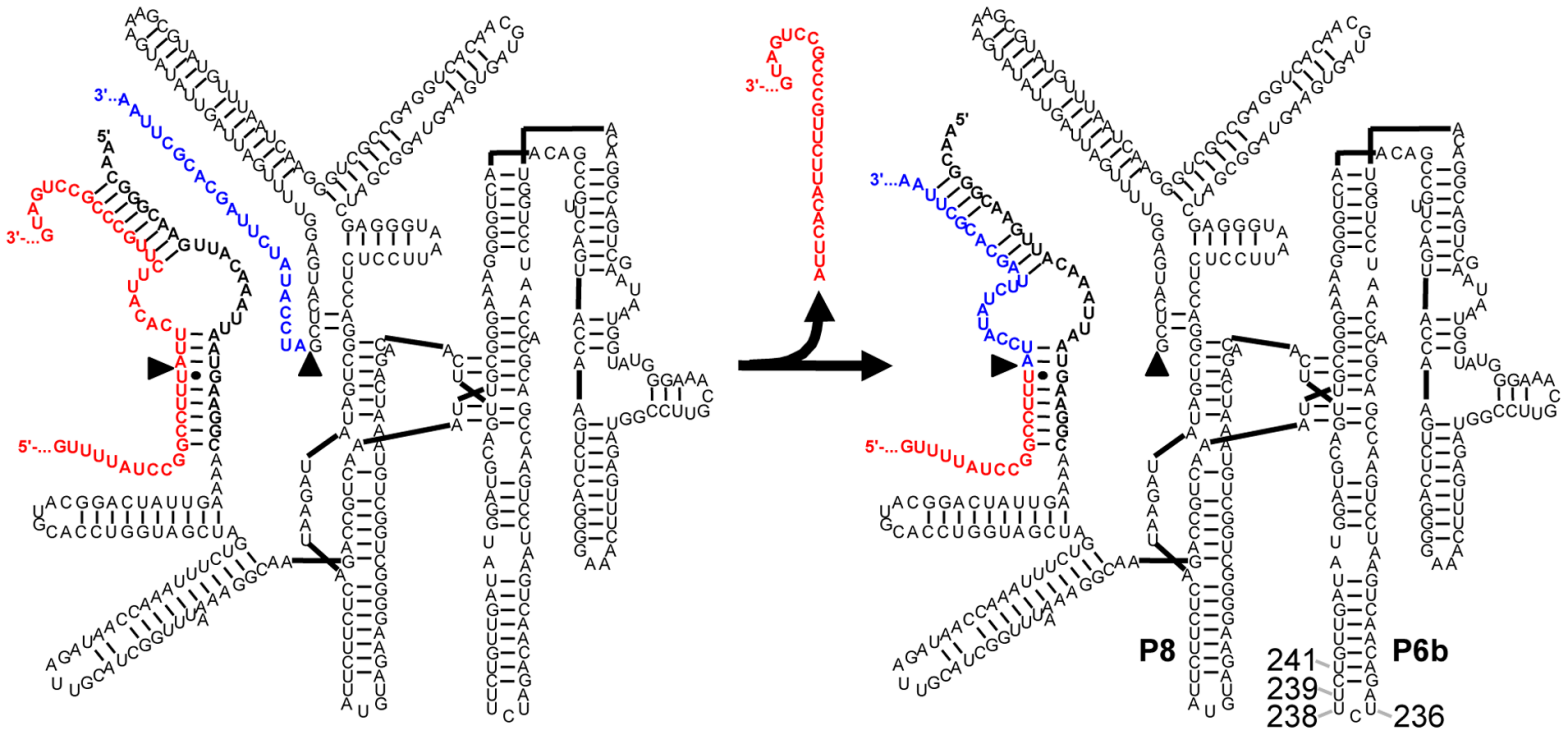
Secondary structure of a trans-splicing group I intron ribozyme. The ribozyme (black) is base-paired to the target site of the mutated *CAT* mRNA (red). The 5′-splice site and 3′-splice site are indicated with arrowheads. During the trans-splicing reaction (arrows from left to right) the 3′-exon of the ribozyme (blue) replaces the 3′-portion of the mutated *CAT* mRNA, leading to the repaired *CAT* mRNA (red/blue). The positions of the P8 and P6b stem-loops in the ribozyme are indicated, together with the four positions 236, 238, 239, and 241.

The model RNA for our evolution was a trans-splicing variant of the group I intron ribozyme from *Tetrahymena* (Figure 1). Group I introns are ribozymes that do not require the spliceosome for their removal from primary transcripts. Instead, they fold into three-dimensional structures that catalyze their own excision and the joining of their flanking exons [11]. These cis-splicing ribozymes have been re-engineered to act in trans, by removing their 5′-exon and replacing it with a short substrate recognition sequence [12]. In this format, the trans-splicing ribozymes specifically recognize a target site on a substrate RNA by base pairing, and replace the 3′-portion of the substrate RNA with their own 3′-exon. In cells, these trans-splicing ribozymes usually repair less than 10% of the target RNAs [12], [13], [14], [15], [16], probably because group I intron ribozymes were evolutionarily optimized for cis-splicing and not for trans-splicing. Indeed, evolving these ribozymes in the lab could increase their efficiencies [10]. Such an evolution of trans-splicing group I intron ribozymes is a good model system to study RNA evolution in cells because in addition to sampling the protein repertoire of the cell, the ribozymes report on a range of functions, such as the formation of a complex three-dimensional structure [17], the recognition of a substrate in trans [12], [18], the catalysis of two transphosphorylation reactions [11], and conformational changes in the RNA structure [19].

To evolve trans-splicing ribozymes in cells it is possible to express large ribozyme libraries in cells, and design the ribozymes such that they can repair the mutated mRNA of chloramphenicol acetyl transferase (CAT) [13] [10]. The CAT enzyme catalyzes the O-acetylation of the antibiotic chloramphenicol, with acetyl-CoA as the acetyl donor [20]. This acetylation renders chloramphenicol unable to inhibit the ribosome, and thereby mediates resistance to chloramphenicol [21]. Upon inactivation of the *CAT* mRNA by a mutation, chloramphenicol resistance is lost. Efficient trans-splicing ribozymes are able to repair the mutation-inactivated *CAT* mRNA in bacterial cells, thereby enabling the bacteria to grow on medium containing chloramphenicol. This allows for the selection of active trans-splicing ribozymes from populations with more than 10^6^ ribozyme variants [13] [10]. Because *selections* contain all sequence diversity in the initial pool, we were interested to see what solutions a ribozyme population could find during *evolution*. This process introduces sequence diversity between multiple selection steps and thereby mimicks more closely the biological evolution of RNAs [2].

Here we show the evolution of a trans-splicing group I intron ribozyme from *Tetrahymena* in *E.coli* cells, for 21 cycles of evolution. The resulting ribozyme population mediated bacterial growth at more than 10-fold higher chloramphenicol concentrations than the parent ribozyme. The focus of this study is to determine how the most efficient, evolved ribozyme was able to achieve high efficiency in cells. The most efficient ribozyme contained four mutations that caused its increased activity. Interestingly, these four mutations did not improve trans-splicing but appear to have recruited the transcription termination factor Rho and improved translation of the repaired mRNAs. These results shed light on how RNAs evolve in cells, by showing that a handful of mutations can be sufficient in an RNA to evolve binding to a protein, and mediate major effects on gene expression.

## Materials and Methods

### In vivo Evolution

The in vivo evolution was conducted [13] essentially as described [10] [22] [23]. In short, a library plasmid was generated from a variant of the plasmid pUC19, by cloning the *CAT* cassette from plasmid pLysS between SphI and HindIII, and the ribozyme expression cassette between BamHI and SacI. This placed the *CAT* gene under the control of a constitutive promoter and the ribozyme cassette under the control of a downregulated version of the IPTG-inducible trc promoter, in which the −30 box was mutated from TTGACA to TTTACA [13], [24]. The *CAT* gene contained a frameshift mutation, the deletion of T273 (position 1 is the A of the ATG start codon). In contrast to published evolution experiments [10], the splice site in the mutated *CAT* mRNA was at position 177 (counted from the A of the ATG translation start codon) and not at position 258. In each cycle of evolution the plasmids were isolated from the selected cells, the ribozyme sequence was amplified by PCR and purified by agarose gel electrophoresis. Mutations or recombination events were introduced into the ribozyme gene during its amplification by mutagenic PCR [22] or the staggered extension process (StEP) [23], respectively. The mutagenesis made use of the lower fidelity of *Taq* DNA polymerase at higher magnesium concentrations and in the presence of 0.5 mM Mn^2+^. The recombination approach used 40 cycles of PCR with annealing times of five seconds and temperature ramping rates of 6°C per second, without extension steps at 72°C. Under these conditions, the PCR primers were incompletely extended in each cycle, dissociated from one template and annealed to a different template in the next cycle for further elongation, thereby facilitating recombination. These recombination conditions were chosen to mediate ∼1 recombination event per ribozyme sequence, on average. The ribozyme gene was then ligated into fresh library plasmid, and ligation products were transformed into electrocompetent *E.coli* cells. The cells were then plated on LB medium containing ampicillin, incubated, washed, and frozen as glycerol stocks. The replating efficiency and the percentage of plasmids containing the ribozyme insert were determined by plating on LB medium containing ampicillin, and colony PCR. The concentration of chloramphenicol in the plates of the selective step of the evolution was adjusted over successive evolution cycles to the fitness of the pool, increasing from 4 µg/mL in the first cycle to 70 µg/mL in the last cycle of the evolution. Because the high mutagenesis rate in the first cycle of evolution (∼7.3 mutations per ribozyme) did not allow mutagenesis in the second cycle (otherwise no viable transformants resulted), cycles 3 to 8 were separated into branch A and branch B, with medium and low mutagenesis rates (4.8 and 2.4 mutations per ribozyme, respectively). Branch A required the lower chloramphenicol concentration of 2 µg/mL to avoid collapse whereas branch B was stable at 4 µg/mL or more. Therefore, the low mutagenesis rate was chosen in later cycles after the material from branches A and B was combined in cycle 9. In each cycle of the evolution, ten ribozyme sequences were obtained to follow the progress of the evolution. Site-directed mutagenesis was employed to generate mutations in the evolved ribozymes, using the QuikChange kit (Stratagene) according to the manufacturer’s instructions.

### Measurement of *E.coli* Growth Rates

The doubling times of *E.coli* cells were determined essentially as described [13], by treating a fresh overnight culture for 1 hour with 1 mM IPTG to induce ribozyme expression, diluting the cells to an OD_600_ of 0.05 in LB medium containing 1 mM IPTG and the appropriate concentration of chloramphenicol, shaking the cells at 37°C, and measuring the increase in the OD_600_. The values of OD_600_ up to 0.6 were fitted to the exponential equation of OD_600_ = a+b•2^∧^(time/c). The parameters a, b, and c were fitted by the least squares method, with the constraints that a≥0 and b≥0.025. The parameter a described non-dividing cells, b described dividing cells, and c corresponded to the doubling time. Doubling times larger than 100 minutes showed large variations between experiments and were therefore described as “>100 minutes”.

### In vitro Trans-splicing Assay

Assays were performed as described previously [18]. In short, ribozymes or the full-length *CAT^MUT^* mRNA were generated by run-off transcription from PCR products and purified by denaturing polyacrylamide gel electrophoresis (PAGE). The ribozyme 3′-exon was truncated at its 3′-terminus so that reaction products could be size separated from substrates and intermediates. Purified *CAT^MUT^* mRNA was 5′-dephosphorylated by Antarctic phosphatase (New England Biolabs) and 5′-radiolabeled with T4 PNK (Invitrogen) and γ[^32^P]ATP. After PAGE purification, trace amounts of radiolabeled products were incubated with 1 µM ribozyme in a buffer containing 1 mM MgCl_2_, 135 mM KCl, 50 mM MOPS/KOH pH 7.0, 20 µM GTP, and 2 mM spermidine at 37°C for 3 hours. Before the reaction, ribozymes were pre-incubated in reaction buffer without magnesium. Reaction products were separated on denaturing 7 M urea 5% PAGE and quantified by phosphorimaging (PMI; Bio-Rad) using the Image Quant software. The percent of repaired *CAT* mRNA was calculated using the signal intensities of substrate, reaction intermediate, and reaction product. The values are the averages from three experiments.

### Measurement of CAT Activity

The CAT activity was measured as described [25] but with 10-fold more cells because the highest levels of CAT activity were ∼2.5-fold below the level resulting from the expression of functional *CAT* mRNA (data not shown). Cells were grown under the same conditions as the assay measuring the growth rate and harvested at an OD_600_ of 0.5. The cells from 2 mL culture were concentrated to 200 µL by centrifugation and frozen. After thawing, 200 µL of 200 mM Tris/HCl pH 7.8 and 10 mM Na_2_EDTA were added, then 4 µL of toluene were added and mixed. Fifteen µL of this solution were mixed with 135 µL of reaction buffer for the final concentrations of 1.0 mM 5,5′-Dithio-bis(2-nitrobenzoic acid) (DTNB), 0.2 mM Acetyl-CoA, and 0.2 mM chloramphenicol. The absorption at 412 nm was measured every 15 seconds in a Nanophotometer (Implen). The slope in the time interval from 6 minutes to 15 minutes was obtained by linear least squares fitting. The units of CAT activity were calculated based on the extinction coefficient 13,600 M^−1^ cm^−1^ for the reaction product 5′-thio-2-nitrobenzoic acid, and the unit definition of CAT activity where one unit catalyzes the acetylation of 1 µmol of chloramphenicol per minute [25].

### Fractionation of Ribosomes

Ribosome fractionations were done as described [26]. 100 mL of cell culture were grown as described above for growth rate measurements. Cells for replicate experiments were from three separate biological samples. The cells were treated with 400 µg/mL chloramphenicol to stall ribosomes and immediately cooled on ice. Cells were lysed using lysozyme (1 mg/mL in 20 mM Tris/HCl pH 7.5, 15 mM MgCl_2_, and 400 µg/mL chloramphenicol) and freeze-thawed, followed by treatment with 0.5% (w/v) sodium deoxycholate. After sedimentation of cell debris and genomic DNA the A_260_ was measured and 180 µg of RNA were loaded on each 10%–40% sucrose gradient (11 mL volume with 20 mM Tris/HCl pH 7.5, 10 mM MgCl_2_, 100 mM NH_4_Cl, and 2 mM β-mercaptoethanol), at 0°C. After centrifugation (3 hours at 260,000×g at 0°C) the gradients were fractionated into 1 mL fractions, with the 70 S peak collected in the first fraction. The observed, increased abundance of RNAs on polysomes was not caused by higher cellular ribosome concentrations because the samples loaded on the sucrose gradients were normalized for their absorption at 260 nm, which is dominated by ribosomal RNA.

### RNA Isolation

RNA was isolated from 100 µL of each fraction of the sucrose gradient, immediately after fractionation of the gradients, using the Nucleospin RNA II kit (Macherey Nagel) according to the manufacturer’s instructions, with on-column DNase digestion. Total RNA was isolated from 2 mL of *E.coli* culture grown logarithmically in LB medium containing 100 µg/mL ampicillin and 1 mM IPTG. Immediately after the OD_600_ had reached 0.5, the cells were pelleted by centrifugation, and the RNA was isolated. RNA isolation from cells was done with the RNAeasy mini kit (Qiagen) according to the manufacturer’s instructions with on-column DNase digestion. For each replicate experiment, three separate biological samples were used to prepare total RNA.

### RT-qPCR

For reverse transcription, 200 ng of total RNA or the RNA corresponding to 13 µL of sucrose gradient (up to 310 ng, as estimated from the A_260_) were used as templates per 20 µL reaction, with Superscript III reverse transcriptase (Invitrogen) according to the manufacturer’s instructions. The reaction products were diluted with water such that the subsequent qPCR quantification cycles were between ∼15 and ∼30 cycles. For gradient fractions these dilutions were 15-fold for ribosomal gradient fractions for *cysG* and *GAPDH*, 50-fold for *CAT* pre-mRNA, ribozyme, and repaired *CAT* mRNA, and 500-fold for 16S rRNA. For total RNA a dilution of 500-fold for all samples gave consistent results. At least two dilutions were tested for each sample, which confirmed the linearity of all assays. Quantitative PCR was performed on the Fast 7500 machine (Applied Biosystems), using the SYBR green qPCR master mix (Applied Biosystems), and an amplification protocol of 95°C/30 seconds, 57°C/30 seconds, and 72°C/30 seconds. In all cases, melting profiles confirmed that specific PCR products were quantitated. The amounts of RNAs were calculated by correlating the quantification cycle value with qPCR from of a plasmid with known concentration (confirming an amplification of about 2-fold per PCR cycle), assuming that a cell density of 1 corresponded to 2 • 10^8^ cells/mL, and assuming that no losses occurred during sample preparations. The RT primer was the same for substrate, ribozyme, and product (5′-CACCGTCTTTCATTGC). The 5′-PCR primers and 3′-PCR primers were 5′-CCGTTCAGCTGGATATTACG and 5′-CATACGGAATTCCGGATGAG (*CAT* pre-mRNA), 5′-AGTGATGCAACACTGGAGCC and 5′-TACTACCGATACGTACACTG (ribozyme), and 5′-CCGTTCAGCTGGATATTACG and 5′-TACTACCGATACGTACACTG (*CAT* mRNA). The silent mutations in the ribozyme 3′-exons were insufficient to rigorously differentiate between *CAT* pre-mRNA and repaired *CAT* mRNA, which was visible in some cross-amplification between samples in pilot experiments (data not shown). Therefore, the RT-qPCR experiments were conducted with a modified ribozyme 3′-exon sequence containing a specifically generated primer binding site, and a complementary 3′-PCR primer. This made it possible to discriminate between mutated *CAT* mRNA and repaired *CAT* mRNA without detectable cross-amplification. Note that this also introduced a stop codon into the 3′-exon but this does not affect the conclusions because the cells were not grown in the presence of chloramphenicol, and because similar results (with some cross-amplification between primers) were obtained when the 3′-primer sites for mutated *CAT* mRNA and repaired *CAT* mRNA differed only in the silent mutations contained in the ribozyme 3′-exon (not shown). Although GAPDH is frequently used as reference RNA in RT-qPCR its reliability has been questioned, therefore we included an additional reference RNA with supposedly more stable expression, *cysG* [27]. The RT primer, 5′-primer, and 3′-primer was 5′-GTTGTCGTACCAGGATAC, 5′- ACTTACGAGCAGATCAAAGC, and 5′- AGTTTCACGAAGTTGTCGTT for GAPDH, repectively. The same primers were 5′-TTAACATGCCTGCATCTG, 5′- TTGTCGGCGGTGGTGATGTC, and 5′- ATGCGGTGAACTGTGGAATAAACG for cysG, respectively. Additionally, 16S rRNA was included to serve as control in the polysome fractionation experiments. For 16S rRNA the RT primer was 5′-GTATTACCGCGGCTGCTG and the 5′- and 3′-PCR primers were 5′-CTCTTGCCATCGGATGTGCCCA and 5′-CCAGTGTGGCTGGTCATCCTCTCA [27].

### Pull-down Experiments with Biotinylated RNA Hairpins

*E.coli* cells were grown in LB medium to an OD_600_ of 0.5, the cells were washed twice in cold 0.2×PBS, and frozen in 1/150 of the cell culture volume. The cells were thawed and suspended in cold 0.2×PBS with 1 mM Na_2_EDTA, 0.1% (w/v) Triton X-100 and 1 mg/mL lysozyme. After 10 minutes incubation on ice the cells were frozen in liquid nitrogen and thawed for five times, then centrifuged. The supernatant was used in the following steps. To 0.3 mg of washed streptavidin-magnetic beads (Promega), 500 pmol of heat-renatured, biotinylated RNA hairpins in 0.2×PBS were added and incubated for 10 minutes on ice. The supernatant was removed from the beads and 660 µL of cell lysate supernatant with 6.6 µL of 300 mM MgCl_2_ were added. After incubation on ice for 10 minutes the beads were washed three times with 0.2×PBS and 0.01% of Triton X-100. Proteins were eluted with LDS sample buffer under heat denaturation (2′/80°C). Samples were run on SDS polyacrylamide gradient gels (4%–12%) and silver stained with the Focus Fast-silver kit (G-Biosciences). Specific bands were excised, destained, and analyzed by the UCSD mass spec facility. Peptides were compared to the *E.coli* database, and only peptides with a confidence of at least 95% were reported. Proteins resulting from sample handling (human keratin and porcine trypsin) were omitted. The GenBank Accession Numbers of the identified proteins are 170083270 (Rho transcription termination factor), 170079798 (protease Do), 170082766 (periplasmic protease), 170080341 (methylthio transferase), 170081402 (succinarginyl dihydrolase), 170083054 (metal dependent hydrolase), 170080578 (inner membrane protein), and 170083396 (glycerol kinase).

## Results

### Evolution of Trans-splicing Ribozymes in Cells

**Figure 2:**
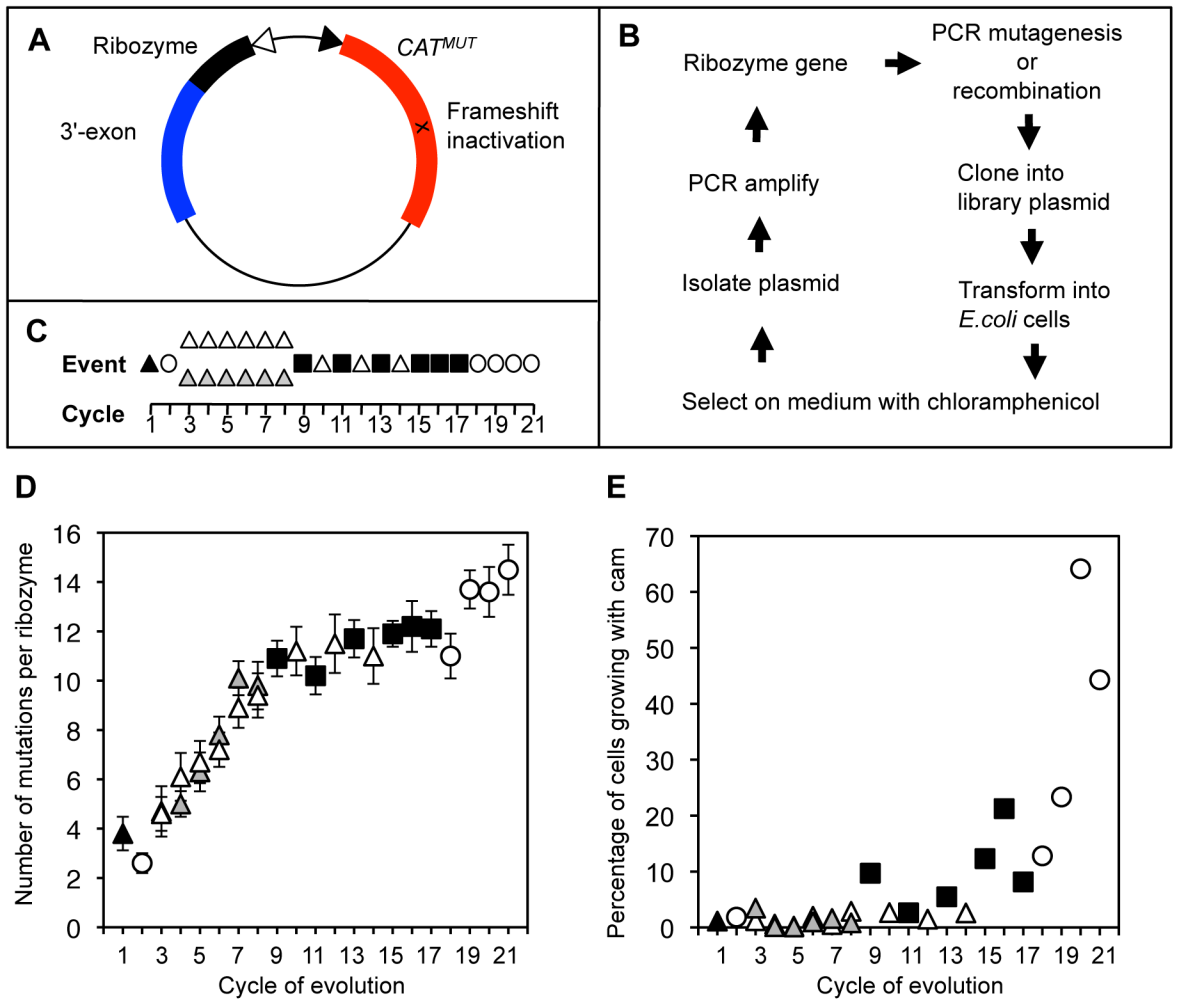
In vivo evolution of trans-splicing ribozymes. *A*, Library plasmid used in the evolution, expressing the mutated *CAT* mRNA (*CAT^MUT^*, red) under the control of a constitutive promoter (filled triangle), with a frameshift mutation (x). The ribozyme (black) is expressed from an inducible promoter (open triangle) and is linked to its 3′-exon (blue). *B*, Flow-chart of events in a single evolution cycle. *C*, Succession of events during the 21 cycles of evolution. Evolutionary cycles with mutagenic PCR are labeled with triangles, cycles with recombination are labeled with filled squares, and enrichment cycles are labeled with empty circles. Empty, grey, and black triangles correspond to a low, medium, and high mutagenesis rate, respectively. *D*, Increase in the number of mutations over the course of the evolution. The average number of mutations per ribozyme sequence is given. Error bars denote the standard error of the mean (n = 10). Symbols are as in (C). *E*, Increase in the activity of the pool during the evolution. The percentage of viable cells growing on plates with 20 µg/mL chloramphenicol is given. Symbols are the same as in (C).

The experimental procedure to evolve trans-splicing variants of the *Tetrahymena* group I intron ribozyme in *E.coli* cells was [13] similar to a previously published procedure [10]. In short, a trans-splicing ribozyme was co-expressed with a mutation-inactivated mRNA of chloramphenicol acetyltransferase (*CAT*) (Figure 2A). The ribozyme’s 5′-terminal targeting region was complementary to a splice site on the mutated *CAT* mRNA, and its 3′-exon was designed to repair the mutated 3′-portion of the *CAT* mRNA. Therefore, efficient ribozymes facilitated the expression of functional CAT enzyme and allowed their host cells to grow on medium containing chloramphenicol (Figure 2B). Mutagenic PCR [28] was used to introduce mutations into the population of ribozyme genes. In each cycle of the evolution, an average of 7 • 10^5^ viable bacterial cells was plated on medium containing chloramphenicol, thereby selecting for ribozymes that worked efficiently in cells. To avoid artifacts based on mutations in the *E.coli* genome or the plasmid, the library plasmids were isolated in each evolutionary cycle from the grown bacterial colonies, and their ribozyme genes were isolated, amplified by PCR with mutagenesis or recombination, purified by agarose gel electrophoresis, and re-cloned into fresh library plasmids. These plasmids were transformed into fresh *E.coli* cells, completing one cycle of the evolution. The used evolution procedure differed from that in our related study [10] by its population sizes, chloramphenicol concentrations, the application of recombination, and the number of evolution rounds.

Starting from a single sequence, the *Tetrahymena* group I intron ribozyme gene was subjected to 21 cycles of evolution (Figure 2C). The selection pressure was adjusted in each cycle of the evolution to the fitness of the evolving population such that an average of 2 • 10^4^ clones (∼3%) formed visible colonies. This allowed building and maintaining population diversity and enriching for increasingly active ribozyme variants. The second cycle of evolution did not use mutagenic PCR because the first cycle used such high mutagenesis that no viable colonies resulted when mutagenesis was included in the second cycle. The following cycles of evolution (cycles 3–8) used two different levels of mutagenesis, a medium level in branch A and a low level in branch B. After cycle 8 the material from both branches was combined and mutagenesis was used only at the low error rate. Note that both branches resulted in the same average increase of 1.1 mutations per ribozyme and per cycle (Figure 2D) despite the different mutagenesis rate. This is only 23% and 46% of the mutations introduced by mutagenic PCR into branch A and branch B, respectively. Therefore, most of the introduced mutations were culled from the population during the selective step, and only a fraction of the introduced mutations ended up increasing the genetic diversity of the evolving ribozyme population.

Recombination was introduced as a PCR-based technique [23] into the evolution procedure starting at cycle 9 (Figure 2C). Our rationale to include recombination was to allow the combination of multiple beneficial mutations from separate ribozyme sequences into one ribozyme sequence, and to allow the removal of deleterious mutations from otherwise efficient ribozymes. The recombination likely did not benefit the accumulation of the four most important, beneficial mutations (see two paragraphs below) because these four mutations occurred within 6 nucleotides, which made it very unlikely that a recombination event occurred between them. To test whether recombination was successful in removing deleterious mutations we compared 40 sequences from cycles 7A, 7B, 8A, and 8B (before recombination) to 40 sequences from cycles 15 to 18 (after recombination). As a measure for deleterious mutations we counted the mutations that occurred in the conserved core of the ribozyme [17], with exception of the P1 helix. This conserved core consisted of 93 nucleotides (G96-G117, C204-U221, A252-G282, C296-A314, and U412-G414). Before the recombination cycles, each set of 10 sequences contained 4.3±1.3 mutations in the conserved core, whereas after the recombination cycles, this value was reduced to 1.0±0.8 mutations. This 4.3-fold reduction of deleterious mutations by recombination is an underestimate because the total number of mutations was 24±10% higher in cycles 15–18 than in cycles 7A, 7B, 8A, and 8B (Figure 2D). These results suggested that the recombination events played an important role in removing deleterious mutations from the evolving ribozyme population.

To enrich for the most efficient ribozymes in the population, the selection pressure was raised after 17 cycles of evolution. This was done by increasing the chloramphenicol concentrations in the selection medium to 70 µg/mL while omitting mutagenesis and recombination. Satisfyingly, the average fitness of the evolving pool increased strongly (Figure 2E).

**Figure 3:**
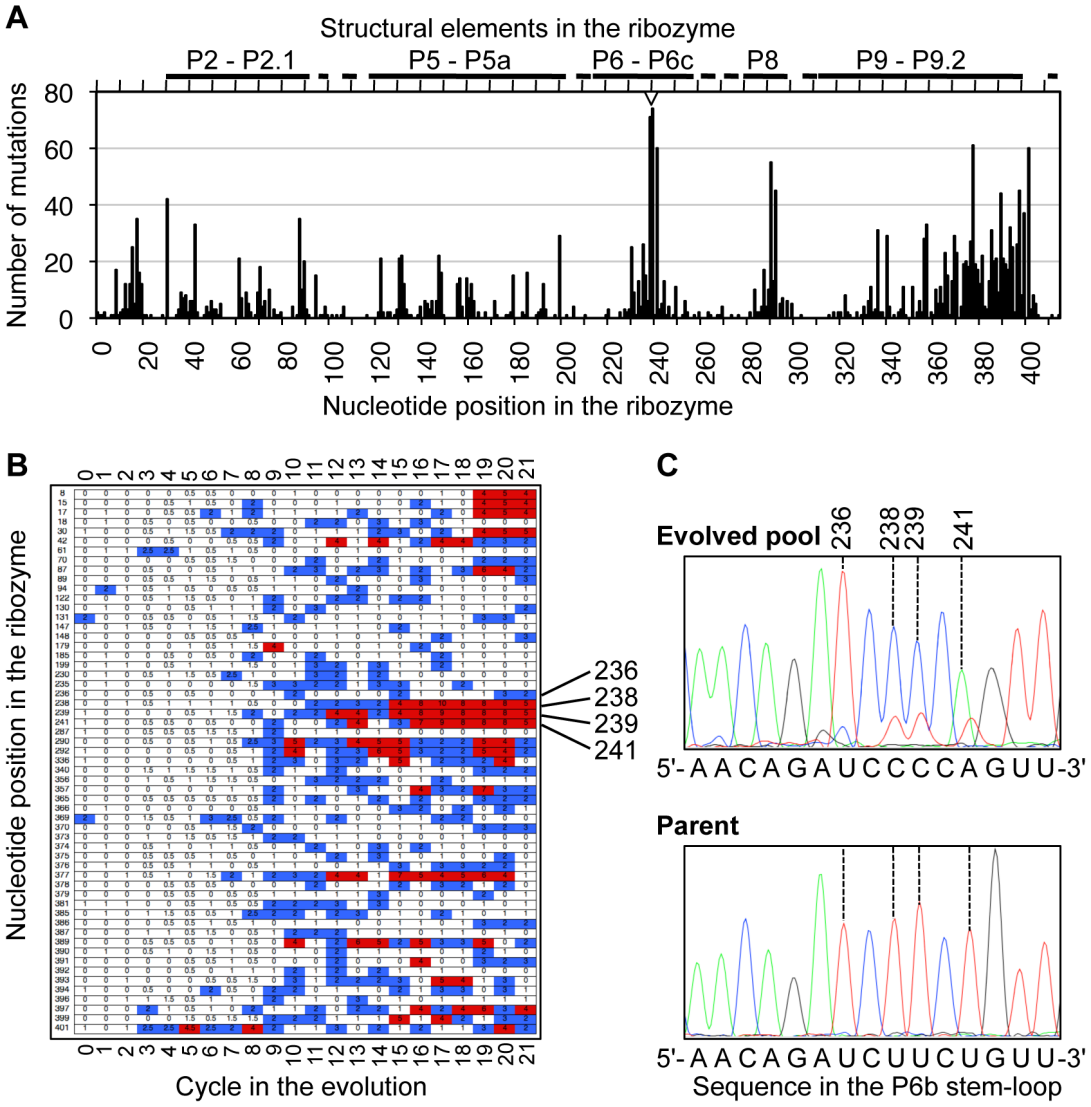
Enrichment of specific mutations during the evolution. *A*, The number of mutations detected over all 21 cycles of evolution is plotted for each of the 414 nucleotide positions in the ribozyme. The graph represents a total of 2438 mutations. The structural elements corresponding to the nucleotide positions according to [51] are given at the top. The short helices P3, P7, and P9.0 are not labeled due to space constraints. The position of the P6b loop is indicated by an empty triangle. *B*, The frequency of mutations that appeared at least 15 times in total is shown over the cycles of evolution. The nucleotide position of these mutations is given in the left-most column. Ten sequences were obtained for each cycle, with blue denoting at least 2 mutations and red denoting at least four mutations detected for that cycle and nucleotide position. For cycles 3–8, where the evolution was split into two branches, the color-coding is averaged over both branches. *C*, The sequencing chromatogram of the evolved plasmid library after cycle 21 shows the enrichment of mutations 238, 239, and 240 in the ribozyme population (top), compared to the parent sequence (bottom). Note that at position 236, “C” (blue) represents only a small proportion of the evolved pool.

The enrichment of specific mutations during the evolution was followed by the analysis of 10 ribozyme sequences in every evolution cycle. Several regions of the ribozyme accumulated many mutations, such as the P8 stem-loop and the P9–P9.2 regions, with the highest frequency of mutations in the P6b stem-loop (Figure 3A). The frequency of these mutations rose during the evolution, most pronounced after evolution cycle 9, where recombination was introduced (Figure 3B). The high frequency of mutations in the P6b stem-loop, namely at positions 236, 238, 239, and 241, was clearly visible in the sequencing chromatogram of the ribozyme pool after 21 cycles of evolution, where three of these four mutations appeared to represent the dominant sequence (Figure 3C).

**Figure 4:**
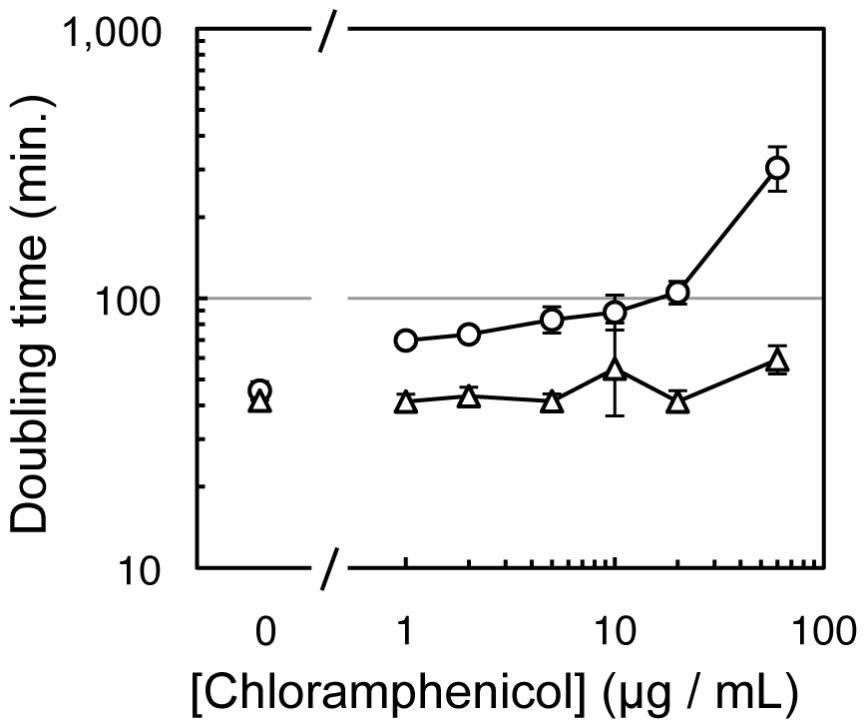
Increased resistance to chloramphenicol in cells expressing the most efficient ribozyme isolated from the evolution. The doubling time of *E.coli* cells in medium containing chloramphenicol is plotted as function of chloramphenicol concentration. The evolved ribozyme (triangles) facilitated shorter doubling times than the parent ribozyme (circles). Note that the horizontal scale is broken to denote the doubling time of both constructs in the absence of chloramphenicol. Error bars denote standard deviations from the geometric means of at least three independent experiments. If error bars are not visible they are smaller than the symbols.

To identify individual ribozyme sequences with high activity the sequences of 30 ribozymes were obtained from these last three cycles of evolution (Figure S1). Comparison of these sequences revealed 15 clones that jointly possessed all mutations that appeared at least twice among the 30 sequences. These 15 clones were individually tested in *E.coli* cells, for their effect on the doubling time in suspension culture in the presence of chloramphenicol. The clone with the highest activity showed cell-doubling times about 2-fold below the cell doubling times with the parent ribozyme, over a wide range of chloramphenicol concentrations (Figure 4). Therefore, this most active ribozyme clone was chosen for further analysis.

### Four Clustered Mutations Mediate High Ribozyme Efficiency in Cells

**Figure 5:**
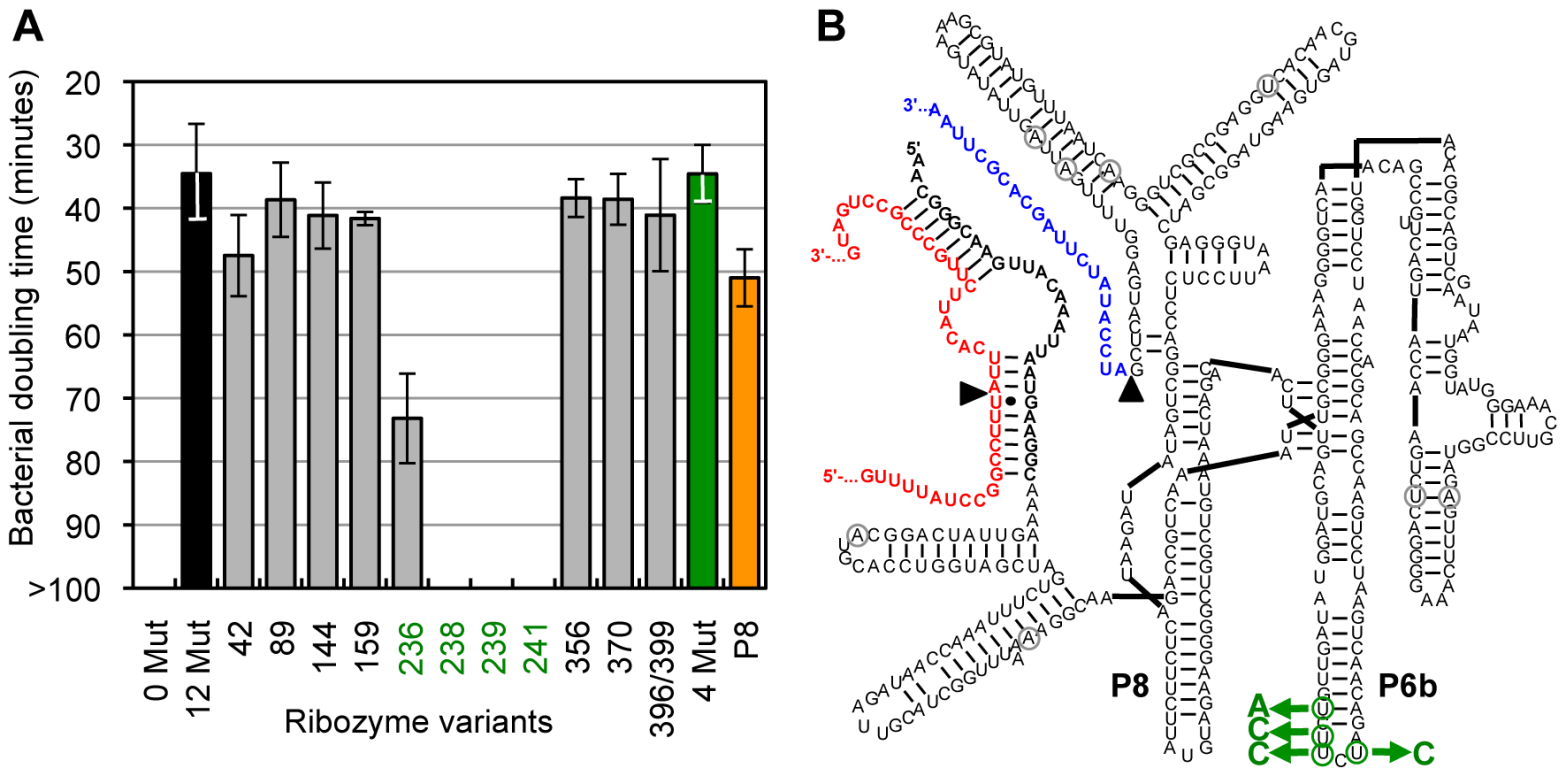
Identification of ribozyme mutations that increase chloramphenicol resistance. *A*, Growth rates of *E.coli* cells containing different ribozyme variants, in medium containing 20 µg/mL chloramphenicol. The parent ribozyme (0 Mut), the most efficient, evolved ribozyme (12 Mut; black), and ribozyme M4 (4 Mut; green) are labeled with the number of mutations distant from the parent ribozyme. The label P8 (orange column) describes the ribozyme where the four mutations were transplanted from the P6b loop to the P8 loop of the parent ribozyme. All other variants (grey columns) are based on the 12-mutation ribozyme and show the effect of individual, labeled reversion mutations to the parent sequence. The absence of a column means that the doubling time was larger than 100 minutes in all experiments. Error bars are standard deviations from at least three experiments. *B*, Secondary structure of the ribozyme with the positions of the 12 mutations in (A) indicated by gray circles. The four beneficial mutations in the P6b stem-loop are shown in green.

The ribozyme sequence with the highest activity in cells contained twelve mutations relative to the parent ribozyme (Figure 5). To identify the mutations that were necessary for the highest activity, we individually reverted each of the 12 mutations to the parent ribozyme sequence and measured the effect of these reversions on the ribozyme activity in cells. Only four of the 12 revertants had decreased activity, thereby identifying the mutations U236C, U238C, U239C, and U241A as necessary for full activity. To test whether these four mutations were also sufficient for full activity, we constructed the ribozyme that differed only in these four mutations from the parent ribozyme sequence, and termed it M4. Indeed, this M4 ribozyme facilitated the same cell doubling time as the most efficient ribozyme from the evolution. Therefore, the four mutations were necessary and sufficient for full activity in vivo. The same four mutations were identified in a similar evolution experiment [10]. However, it was unknown how these four mutations were able to mediate higher antibiotic resistance of the cells.

Interestingly, all four mutations of M4 were positioned in the P6b stem-loop (Figure 5). These positions are exposed to the solvent and distant from the catalytic site of the ribozyme [29], [30]. To investigate whether these mutations acted intramolecularly (e.g. by aiding folding) or intermolecularly (e.g. by binding to a cellular factor) we transplanted the sequence of the mutated P6b stem-loop to the P8 stem-loop. The P8 stem-loop appeared to be a good target for this transplantation because like the P6b stem-loop, the P8 stem-loop is distant from the active site, not involved in tertiary interactions, and solvent-exposed [29]. The results showed that the ribozyme with the evolved mutations in the P8 loop was similarly efficient in cells, demonstrating that the precise position of the mutated stem-loop was not crucial for its activity. These results suggested that the mutations did not act intramolecularly (e.g. by aiding folding) but served to interact with a cellular factor.

### The Four Evolved Mutations Facilitate Protein Binding

**Figure 6:**
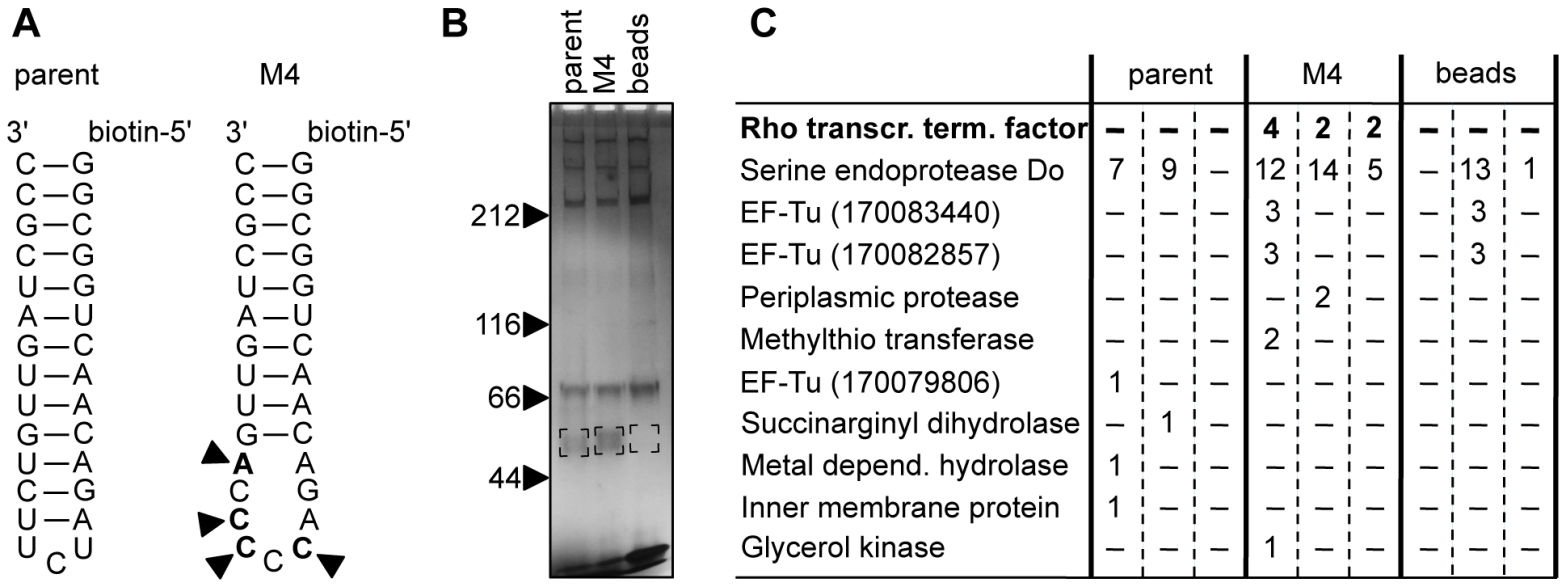
Identification of an *E.coli* protein that specifically interacts with the evolved P6b stem-loop. *A*, Secondary structure of two 5′-biotinylated RNA hairpins that were used in the pull-down experiments. The four evolved, beneficial mutations of M4 are labeled with arrowheads. *B*, Silverstained SDS-polyacrylamide gel with proteins that were pulled down by the biotinylated hairpins. The size of marker proteins in kDa is indicated on the left. The area of the excised gel fragments at ∼50 kDa is indicated by black corners. *C*, Proteins that were identified by mass spectrometry from the excised gel fragments. Given is the number of peptides that were identified for the respective proteins with at least 95% confidence. The three columns for each experiment (parent, M4, beads) show the results from experiments with three biological replicates. Results for the transcription termination factor Rho are shown in bold.

To identify a cellular factor that bound to the evolved P6b stem-loop we performed pull-down experiments with lysates from *E.coli* cells (Figure 6). The pull-down experiments utilized biotinylated RNA stem-loops that contained the sequence of the parent P6b stem-loop or that of the M4 P6b stem-loop, with four additional base pairs to stabilize the helices (Figure 6A). The proteins that were pulled down via the RNA stem-loops were separated by denaturing SDS-polyacrylamide gels, visualized by silver staining (Figure 6B), and identified by Mass Spectrometry (Figure 6C). The transcription termination factor Rho was the only protein that preferentially interacted with the M4 hairpin compared to the parent hairpin, in three independent experiments.

The known RNA binding specificity of Rho fits well with the C-rich sequence of the evolved P6b stem-loop (Figure 6A): Rho is known to bind oligo(C) sequences [31], and the mutations in the evolved P6b stem-loop generated a (C)_5_ sequence. When we extended the oligo(C) sequence in the P6b stem-loop by mutating 5′-AGACCCCCA-3′ of the M4 ribozyme to 5′-ACCCCCCCA-3′ and 5′-CCCCCCCCC-3′ it resulted in the same cell doubling times as M4 (34±2 minutes for the M4 ribozyme and 35±1 minutes and 36±1 minutes for the mutants, respectively). These results were consistent with the model that the characteristic of a high C-content in the P6b stem-loop of the M4 ribozyme mediated the recruitment of Rho.

### Effects of the Four Evolved Mutations on Ribozyme Function

**Figure 7:**
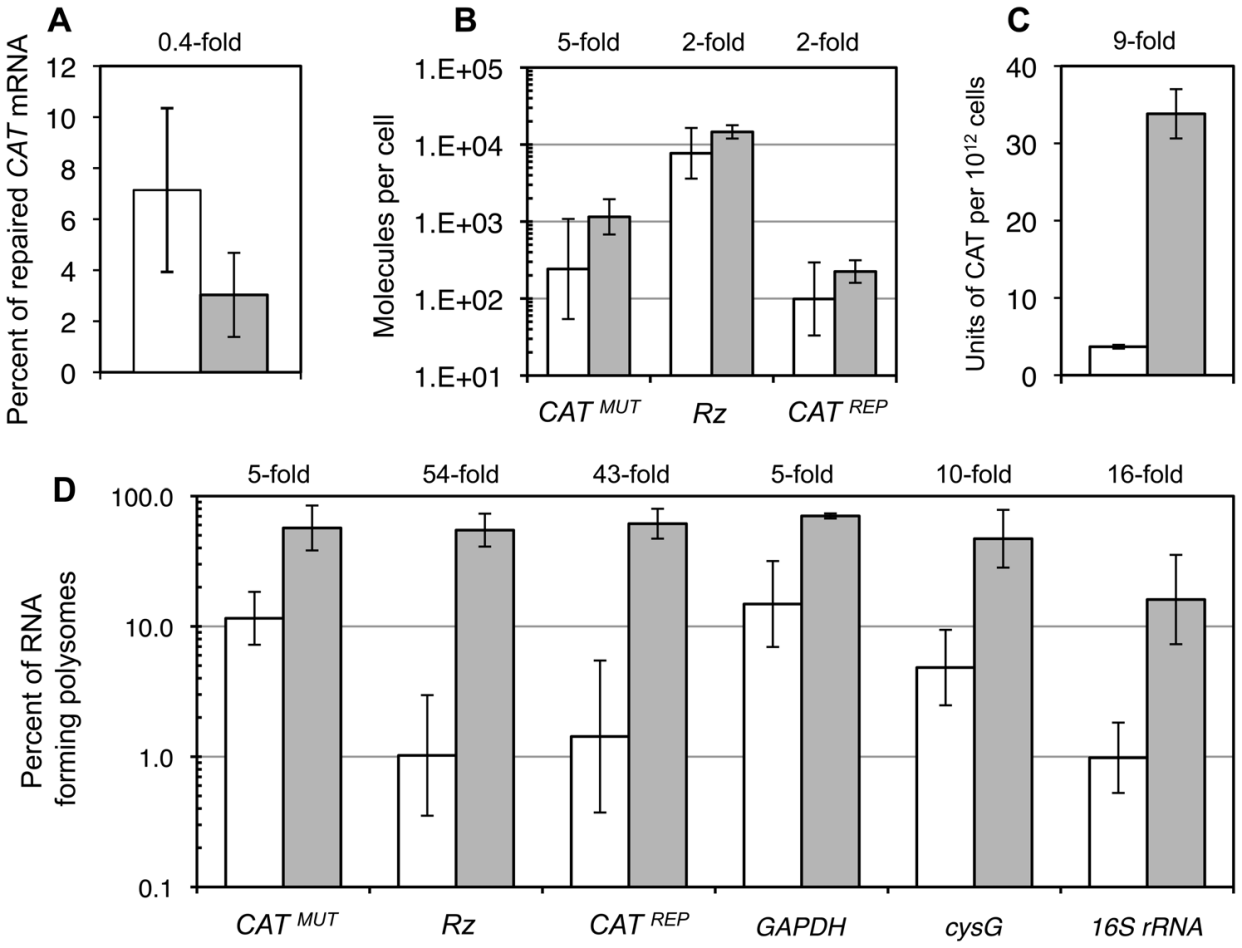
Effects of the M4 mutations on ribozyme function in vitro and in vivo. The values on top of the graphs show the -fold increase of activity from parent ribozyme (white columns) to the M4 ribozyme (gray columns). *A,* Trans-splicing efficiency in vitro. The graph shows the percent of *CAT^MUT^* RNA repaired within 3 hours of incubation. *B*, Trans-splicing efficiency in cells. Shown is the number of molecules of *CAT^MUT^* RNA, ribozyme (Rz), and *CAT^REP^* RNA per *E.coli* cell, as calculated from RT-qPCR experiments with total RNA. *C*, Enzymatic activity of CAT enzyme in *E.coli* cell lysates. Error bars are standard deviations from three biological replicates. *D*, Abundance of RNAs on ribosomes from *E.coli* strains expressing the parent ribozyme (white columns) or the ribozyme with four mutations M4 (grey columns). Shown is the percentage of RNAs detected in all fractions of the sucrose gradient, that were found in the polysome fractions. The -fold values above the graph show the increased loading of the respective RNA on polysomes for the M4 ribozyme versus the parent ribozyme. Error bars show standard deviations from three biological replicates.

To investigate how the four mutations in the M4 ribozyme increased ribozyme efficiency in cells we measured the effects of the mutations on ribozyme function in vitro and in vivo (Figure 7). The in vitro trans-splicing activity of the M4 ribozyme was not increased over the parent ribozyme (Figure 7A). We were surprised to find that the amount of repaired *CAT* mRNA was also not significantly increased in cells with the M4 ribozyme relative to the parent ribozyme, as judged by RT-qPCR analysis of total RNA from cells (Figure 7B). In contrast, the CAT enzyme activity in cell lysate was increased by 9-fold, in cells with the M4 ribozyme relative to the parent ribozyme (Figure 7C). This suggested that the M4 ribozyme led to more efficient translation of the trans-spliced *CAT* mRNA because the cells containing the M4 ribozyme generated 9-fold higher CAT enzyme activity than the parent ribozyme, from similar levels of trans-spliced *CAT* mRNA.

To test whether the M4 ribozyme led to a more efficient recruitment of ribosomes to the *CAT* mRNA we isolated ribosomes from *E.coli* cells and fractionated them on sucrose gradients into 70 S fractions and polysome fractions (Figure S2). Efficiently translated RNAs were expected to be associated more with polysomes, whereas inefficiently translated RNAs were expected to be associated more with single ribosomes. To determine which RNA was associated with polysomes or single ribosomes we isolated the RNA from these fractions and measured the concentration of mutated *CAT* mRNA, ribozyme, repaired *CAT* mRNA, 16S rRNA, and two control mRNAs (*GAPDH* and *cysG*) (Figure S3). The percentage of RNA associated with polysomes compared to single ribosomes was between 5-fold and 55-fold higher in cells with the M4 ribozyme than in cells with the parent ribozyme (Figure 7D). The association of the ribozyme and the repaired *CAT* mRNA with polysomes was increased ∼50-fold, and the association of other mRNAs with polysomes was increased 5- to 10-fold. The percentage of 16S rRNA on polysomes was increased at the intermediate level of 16-fold. This suggested that the M4 mutation caused a non-specific upregulation of polysome assembly in the *E.coli* cells. These results - a weaker, non-specific upregulation of polysome assembly for several unrelated mRNAs and 16S rRNA, and a stronger, specific assembly of polysomes on *CAT* mRNA - support the interpretation that an increase in translation efficiency is the reason why the four mutations in the M4 ribozyme lead to 9-fold higher levels of CAT enzyme from similar levels of the repaired *CAT* mRNA.

## Discussion

This study describes the experimental evolution of a trans-splicing group I intron ribozyme for increased efficiency in *E.coli* cells. Four mutations mediated the activity increase of the most efficient, evolved ribozyme. The mutations created a (C)_5_ sequence in the P6b stem-loop of the ribozyme, which facilitated binding to the protein Rho, and caused a widespread effect on gene expression in *E.coli* cells. The 9-fold increase in activity of the gene product of the targeted mRNA, chloramphenicol acetyl transferase (CAT), appeared to be caused not by an increase in trans-splicing activity but by an increase in translation of the trans-spliced *CAT* mRNA.

Because the four evolved ribozyme mutations facilitated the binding of Rho, understanding the function of Rho is important to understand why the four ribozyme mutations were beneficial. The biological function of Rho is transcription termination (for recent overviews see [32], [33], [34], [35]). Rho forms circular homohexamers that fluctuate between an open and a closed conformation. Upon binding to the C-rich *rut* sites on mRNA, the Rho hexamers close, with the mRNA threaded through the center of the donut-shaped hexamer [34], [36], [37], [38]. The ATP dependent motor of Rho then forces the Rho hexamer to migrate along the mRNA in 5′- to 3′-direction [34], [39]. Because this process is co-transcriptional, Rho can catch up with the transcription elongation complex (TEC) when the TEC pauses. Upon reaching the TEC the helicase activity of Rho separates the nascent RNA transcript from the DNA transcription bubble [40], [41], thereby terminating transcription. This function of Rho takes place on a genome-wide scale to match transcription with translational needs [35].

How could the interaction between the evolved M4 ribozyme and the transcription termination factor Rho cause the observed effects on gene expression in *E.coli*? Rho is known to have a micromolar affinity towards (C)_7_ and (C)_8_ [31], and poly(C) can trap Rho in a state that is termination inactive [42]. Therefore, it appears plausible that the (C)_5_ sequence in the M4 ribozymes reduced Rho mediated transcription termination activity in *E.coli* cells.

The apparent effect of the M4 mutations on the presence on polyribosomes was about 50-fold for the repaired *CAT* mRNA and ribozyme that probably stayed associated with repaired *CAT* mRNA and 5- to 10-fold for unrelated RNAs (see the 5–10-fold effect on *GAPDH* and *cysG* in figure 7). A nonspecific reduction of Rho activity by the M4 mutations would be possible because the estimated 17,000 M4 ribozyme molecules per cell (Figure 7B) outnumbered the ∼5,000 Rho *monomers* per cell (Rho contributes 0.1–0.15% to the *E.coli* cell protein [43] and each cell has ∼340 fg of protein [44]). This interpretation of the non-specific effect is also consistent with the widespread effect of Rho on *E.coli* gene expression [35]. In contrast, the stronger, specific effect of the M4 ribozyme on repaired *CAT* mRNA (∼50-fold, see figure 7) is caused by the specific co-localization of the ribozyme with *CAT* mRNA: The M4 ribozyme is targeted to its splice site on the emerging *CAT* mRNA, and the resulting higher concentration of the (C)_5_ sequence of the M4 ribozyme would preferentially inhibit Rho molecules near *CAT* mRNA. In summary, we propose that the difference in strength between the non-specific effect (5–10-fold) and the specific effect (∼50-fold), on the RNA presence on polysomes was caused by the localization of the M4 ribozyme on the *CAT* mRNA but not unrelated mRNAs. It is currently unclear what exact mechanism could be used by Rho to modulate the assembly of polysomes. Hypotheses for such a mechanism could be based on Rho’s transcription terminator function, the observation that translation in *E.coli* is co-transcriptional [45], and the finding that Rho coordinates transcription with translation on a genome-wide level [35].

**Figure 8:**
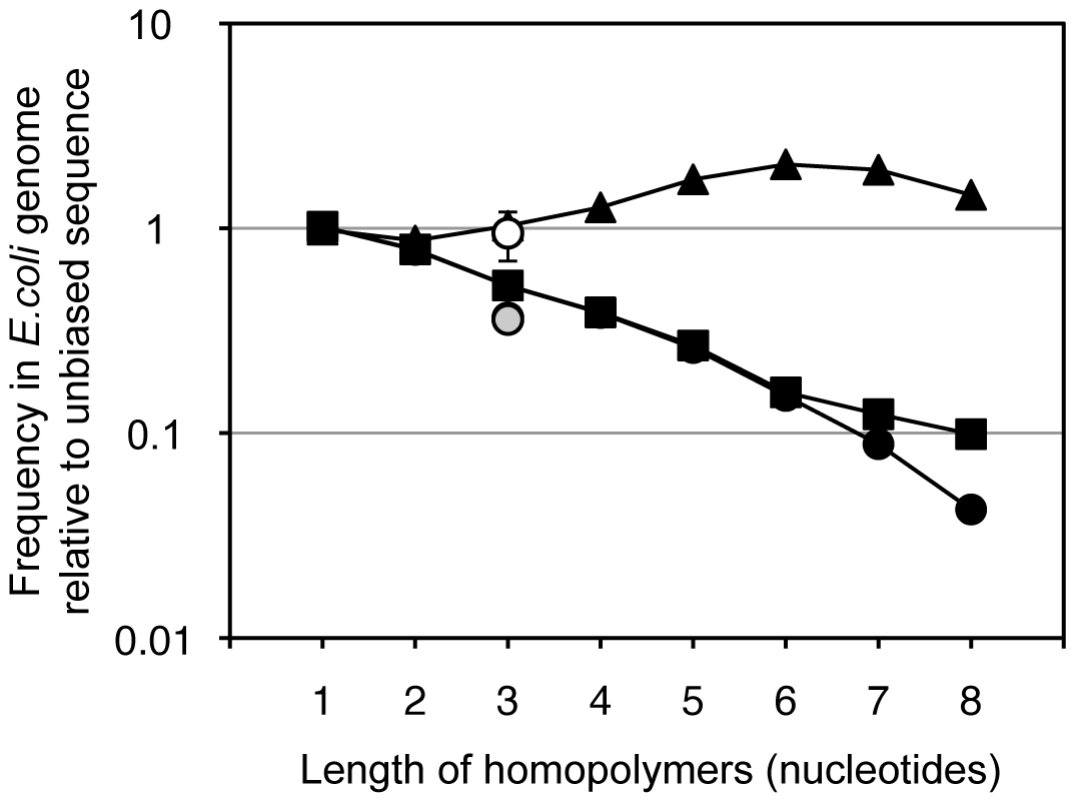
Frequency of oligo(C) sequences in the genome of *E.coli*. The graph shows the relative abundance of homopolymers in the genome of *E.coli*, as a function of the length of the homopolymer sequence. Note that the values for oligo(C) (filled, black squares) and oligo(G) (filled, black circles) show the frequency of oligo(C) on the top and bottom strand of the genome, respectively. The values for oligo(A) and oligo(T) (filled triangles) are so similar that they cannot be distinguished in this graph. As comparison, the average of all triplet codons (white circle with standard deviation as error bars) and the frequency of the stop codon TAG and its reverse complement CTA (circles with grey filling) are shown. The genome used for this analysis was from *E.coli* K12 sub-strain MG1655 (gi|49175990).

Because the (C)_5_ sequence in the P6b stem-loop of the M4 ribozyme had such a profound effect on gene expression in *E.coli* we hypothesized that this effect would exert a strong selection pressure on the abundance of oligo(C) sequences in the *E.coli* genome. Indeed, (C)_n_ homopolymers are strongly underrepresented in the *E.coli* genome (Figure 8). The sequences (C)_5_, (C)_6_, and (C)_7_ are represented only at 27%, 16%, and 12% of the value expected from an unbiased distribution. This effect is not caused by a nucleotide bias in the *E.coli* genome, which contains 25.4% C and 25.37% G. The underrepresentation is even visible at the level of triplet sequences, where CCC and its reverse complement GGG are present only at 53% and 52% of their expected frequencies, respectively. Only the triplets TAG and its reverse complement CTA are represented less (both at 36%), perhaps due to the role of TAG as stop codon. This strong underrepresentation of oligo(C) in the *E.coli* genome suggests that (C)_n_ oligomers have a biological role. This role may be the interference with Rho, consistent with the postulated role of the evolved (C)_5_ sequence in the M4 ribozyme (see above).

The mutations evolved in the P6b stem-loop showed a similar effect in cells when they were transplanted to the P8 stem-loop (Figure 5A). Therefore, one might wonder why the same activity-mediating mutations did not evolve in the P8 stem-loop. The answer may lie in the sequence of the stem-loops and the frequency of different mutation events in the used method of mutagenic PCR [28]. The evolution of the P6 stem-loop from the sequence 5′-AGAUCUUCU-3′ to 5′-AGACCCCCA-3′ required three U to C transitions and one U to A transversions. These two mutations were the two most frequent mutations we found among the six possible transitions and transversions. In contrast, the P8 stem-loop 5′-GAUGUAUUC-3′ would most likely have arrived at the sequence 5′-GAUCCCCCA-3′, in order to widen the loop and generate a (C)_5_ sequence in the same position. The six mutations required for this change would have included not only four frequent mutations (one G to A, three U to C) but also two rare mutations (one A to C and one G to C). Therefore, the (C)_5_ sequence would have been much less likely to evolve in the P8 stem-loop.

The evolution of a ribozyme population in cells presented here and in a related study [10] is different from previous selection studies [46], [47], [48], [49], [50], because *evolution* introduces sequence diversity over multiple selection steps, whereas *selection* introduces sequence diversity only in the initial library [2]. Five previous studies selected ribozymes from ribozyme libraries in cells. (i) Variants of the cis-splicing group I intron ribozyme from *Tetrahymena* were selected in *E.coli* cells, and showed improved splicing due to mutations in the P1 stem [48]. (ii) The selection of trans-splicing group I intron ribozymes from *Tetrahymena* identified the fittest ribozymes from a library of thirteen variants, thereby establishing a selection system in yeast cells [47]. (iii) Optimal hammerhead ribozyme cleavage sites in HIV-1 RNA were determined by randomizing the ribozyme’s target recognition sequence and selecting the most efficient ribozymes in human embryonic kidney cells [49]. (iv) The linker region between hammerhead ribozymes and a theophylline aptamer was optimized from a library of 64 different linker sequences, in *E.coli* cells [50]. (v) The stem II and loop II sequences of a self-cleaving hammerhead ribozyme were optimized by the selection from 70,000 sequence variants in human HeLa cells [46]. These five studies led to ribozymes with improved folding and/or target binding characteristics, but none of them resulted in the recruitment of a cellular protein. In contrast, the evolution in the present study facilitated the binding of a protein by the ribozyme. Therefore, the introduction of mutations over many cycles of evolution, which mimicks more closely the natural evolution of RNAs, may be an important factor in the evolution of RNA-protein interactions.

## Supporting Information

Figure S1 Mutations in the 30 ribozymes isolated from the three last cycles of the evolution (cycles 19, 20, and 21).
(TIF)

Figure S2 Polyribosome analysis. Shown is the absorption at 254 nm during the fractionation of ribosomes and polyribosomes on a representative sucrose gradient, and the abundance of RNAs in gradienmt fractions as judged by RT-qPCR.
(TIF)

Figure S3 Abundance of RNAs on ribosomes from *E. coli* strains expressing the parent ribozyme or the M4 ribozyme. The percentage of RNAs on polysomes is given. Data are shown for the mutated, primary transcript of *CAT* mRNA, the ribozyme, the repaired *CAT* mRNA, *16S rRNA*, and the mRNAs of *GAPDH* and *cysG*.
(TIF)

## References

[1] Dobzhansky T (1964) Biology, Molecular and Organismic. Am Zool 4: 443–452. 14223586 10.1093/icb/4.4.443

[2] Beaudry AA, Joyce GF (1992) Directed evolution of an RNA enzyme. Science 257: 635–641. 1496376 10.1126/science.1496376

[3] Lehman N, Joyce GF (1993) Evolution in vitro: analysis of a lineage of ribozymes. Curr Biol 3: 723–734. 11539560 10.1016/0960-9822(93)90019-k

[4] Burton AS, Lehman N (2006) Calcium(II)-dependent catalytic activity of the Azoarcus ribozyme: testing the limits of resolution for in vitro selection. Biochimie 88: 819–825. 16494986 10.1016/j.biochi.2006.01.010

[5] Hayden EJ, Ferrada E, Wagner A (2011) Cryptic genetic variation promotes rapid evolutionary adaptation in an RNA enzyme. Nature 474: 92–95. 21637259 10.1038/nature10083

[6] Hayden EJ, Wagner A (2012) Environmental change exposes beneficial epistatic interactions in a catalytic RNA. Proc Biol Sci 279: 3418–3425. 22719036 10.1098/rspb.2012.0956 PMC3396916

[7] Hayden EJ, Weikert C, Wagner A (2012) Directional selection causes decanalization in a group I ribozyme. PLoS One 7: e45351. 23028955 10.1371/journal.pone.0045351 PMC3445466

[8] Carothers JM, Oestreich SC, Davis JH, Szostak JW (2004) Informational complexity and functional activity of RNA structures. J Am Chem Soc 126: 5130–5137. 15099096 10.1021/ja031504a PMC5042360

[9] Salehi-Ashtiani K, Szostak JW (2001) In vitro evolution suggests multiple origins for the hammerhead ribozyme. Nature 414: 82–84. 11689947 10.1038/35102081

[10] Amini ZN, Muller UF (2013) Low selection pressure AIDS the evolution of cooperative ribozyme mutations in cells. J Biol Chem 288: 33096–33106. 24089519 10.1074/jbc.M113.511469 PMC3829158

[11] Kruger K, Grabowski PJ, Zaug AJ, Sands J, Gottschling DE, et al (1982) Self-splicing RNA: autoexcision and autocyclization of the ribosomal RNA intervening sequence of Tetrahymena. Cell 31: 147–157. 6297745 10.1016/0092-8674(82)90414-7

[12] Sullenger BA, Cech TR (1994) Ribozyme-mediated repair of defective mRNA by targeted, trans-splicing. Nature 371: 619–622. 7935797 10.1038/371619a0

[13] Olson KE, Muller UF (2012) An in vivo selection method to optimize trans-splicing ribozymes. RNA 18: 581–589. 22274958 10.1261/rna.028472.111 PMC3285944

[14] Byun J, Lan N, Long M, Sullenger BA (2003) Efficient and specific repair of sickle beta-globin RNA by trans-splicing ribozymes. RNA 9: 1254–1263. 13130139 10.1261/rna.5450203 PMC1370489

[15] Rogers CS, Vanoye CG, Sullenger BA, George AL Jr (2002) Functional repair of a mutant chloride channel using a trans-splicing ribozyme. J Clin Invest 110: 1783–1789. 12488428 10.1172/JCI200216481 PMC151654

[16] Shin KS, Sullenger BA, Lee SW (2004) Ribozyme-mediated induction of apoptosis in human cancer cells by targeted repair of mutant p53 RNA. Mol Ther 10: 365–372. 15294183 10.1016/j.ymthe.2004.05.007

[17] Michel F, Westhof E (1990) Modelling of the three-dimensional architecture of group I catalytic introns based on comparative sequence analysis. J Mol Biol 216: 585–610. 2258934 10.1016/0022-2836(90)90386-Z

[18] Meluzzi D, Olson KE, Dolan GF, Arya G, Muller UF (2012) Computational prediction of efficient splice sites for trans-splicing ribozymes. RNA 18: 590–602. 22274956 10.1261/rna.029884.111 PMC3285945

[19] Shi X, Mollova ET, Pljevaljcic G, Millar DP, Herschlag D (2009) Probing the dynamics of the P1 helix within the Tetrahymena group I intron. J Am Chem Soc 131: 9571–9578. 19537712 10.1021/ja902797j PMC2758093

[20] Shaw WV (1967) The enzymatic acetylation of chloramphenicol by extracts of R factor-resistant Escherichia coli. J Biol Chem 242: 687–693. 5335032

[21] Tritton TR (1979) Ribosome-chloramphenicol interactions: a nuclear magnetic resonance study. Arch Biochem Biophys 197: 10–17. 543710 10.1016/0003-9861(79)90212-1

[22] Cadwell RC, Joyce GF (1994) Mutagenic PCR. PCR Methods Appl 3: S136–140. 7920233 10.1101/gr.3.6.s136

[23] Zhao H, Giver L, Shao Z, Affholter JA, Arnold FH (1998) Molecular evolution by staggered extension process (StEP) in vitro recombination. Nat Biotechnol 16: 258–261. 9528005 10.1038/nbt0398-258

[24] Weiss DS, Chen JC, Ghigo JM, Boyd D, Beckwith J (1999) Localization of FtsI (PBP3) to the septal ring requires its membrane anchor, the Z ring, FtsA, FtsQ, and FtsL. J Bacteriol 181: 508–520. 9882665 10.1128/jb.181.2.508-520.1999 PMC93405

[25] Shaw WV (1975) Chloramphenicol acetyltransferase from chloramphenicol-resistant bacteria. Methods Enzymol 43: 737–755. 1094240 10.1016/0076-6879(75)43141-x

[26] Shi X, Chiu K, Ghosh S, Joseph S (2009) Bases in 16S rRNA important for subunit association, tRNA binding, and translocation. Biochemistry 48: 6772–6782. 19545171 10.1021/bi900472a PMC2782751

[27] Zhou K, Zhou L, Lim Q, Zou R, Stephanopoulos G, et al (2011) Novel reference genes for quantifying transcriptional responses of Escherichia coli to protein overexpression by quantitative PCR. BMC Mol Biol 12: 18. 21513543 10.1186/1471-2199-12-18 PMC3110127

[28] Cadwell RC, Joyce GF (1992) Randomization of genes by PCR mutagenesis. PCR Methods Appl 2: 28–33. 1490172 10.1101/gr.2.1.28

[29] Lehnert V, Jaeger L, Michel F, Westhof E (1996) New loop-loop tertiary interactions in self-splicing introns of subgroup IC and ID: a complete 3D model of the Tetrahymena thermophila ribozyme. Chem Biol 3: 993–1009. 9000010 10.1016/s1074-5521(96)90166-0

[30] Guo F, Gooding AR, Cech TR (2004) Structure of the Tetrahymena ribozyme: base triple sandwich and metal ion at the active site. Mol Cell 16: 351–362. 15525509 10.1016/j.molcel.2004.10.003

[31] Wang Y, von Hippel PH (1993) Escherichia coli transcription termination factor rho. II. Binding of oligonucleotide cofactors. J Biol Chem 268: 13947–13955. 8314761

[32] Nudler E, Gottesman ME (2002) Transcription termination and anti-termination in E. coli. Genes Cells 7: 755–768. 12167155 10.1046/j.1365-2443.2002.00563.x

[33] Peters JM, Mooney RA, Kuan PF, Rowland JL, Keles S, et al (2009) Rho directs widespread termination of intragenic and stable RNA transcription. Proc Natl Acad Sci U S A 106: 15406–15411. 19706412 10.1073/pnas.0903846106 PMC2741264

[34] Skordalakes E, Berger JM (2006) Structural insights into RNA-dependent ring closure and ATPase activation by the Rho termination factor. Cell 127: 553–564. 17081977 10.1016/j.cell.2006.08.051

[35] Cardinale CJ, Washburn RS, Tadigotla VR, Brown LM, Gottesman ME, et al (2008) Termination factor Rho and its cofactors NusA and NusG silence foreign DNA in E. coli. Science 320: 935–938. 18487194 10.1126/science.1152763 PMC4059013

[36] Richardson LV, Richardson JP (1996) Rho-dependent termination of transcription is governed primarily by the upstream Rho utilization (rut) sequences of a terminator. J Biol Chem 271: 21597–21603. 8702947 10.1074/jbc.271.35.21597

[37] Gogol EP, Seifried SE, von Hippel PH (1991) Structure and assembly of the Escherichia coli transcription termination factor rho and its interaction with RNA. I. Cryoelectron microscopic studies. J Mol Biol 221: 1127–1138. 1719215 10.1016/0022-2836(91)90923-t

[38] Yu X, Horiguchi T, Shigesada K, Egelman EH (2000) Three-dimensional reconstruction of transcription termination factor rho: orientation of the N-terminal domain and visualization of an RNA-binding site. J Mol Biol 299: 1279–1287. 10873452 10.1006/jmbi.2000.3810

[39] Walstrom KM, Dozono JM, von Hippel PH (1997) Kinetics of the RNA-DNA helicase activity of Escherichia coli transcription termination factor rho. 2. Processivity, ATP consumption, and RNA binding. Biochemistry 36: 7993–8004. 9201946 10.1021/bi963180r

[40] Brennan CA, Dombroski AJ, Platt T (1987) Transcription termination factor rho is an RNA-DNA helicase. Cell 48: 945–952. 3030561 10.1016/0092-8674(87)90703-3

[41] Brennan CA, Steinmetz EJ, Spear P, Platt T (1990) Specificity and efficiency of rho-factor helicase activity depends on magnesium concentration and energy coupling to NTP hydrolysis. J Biol Chem 265: 5440–5447. 1690711

[42] Walstrom KM, Dozono JM, Robic S, von Hippel PH (1997) Kinetics of the RNA-DNA helicase activity of Escherichia coli transcription termination factor rho. 1. Characterization and analysis of the reaction. Biochemistry 36: 7980–7992. 9201945 10.1021/bi963179s

[43] Imai M, Shigesada K (1978) Studies on the altered rho factor in a nitA mutants of Escherichia coli defective in transcription termination. I. Characterization and quantitative determination of rho in cell extracts. J Mol Biol 120: 451–466. 148514 10.1016/0022-2836(78)90348-0

[44] Cox RA (2003) Correlation of the rate of protein synthesis and the third power of the RNA : protein ratio in Escherichia coli and Mycobacterium tuberculosis. Microbiology 149: 729–737. 12634341 10.1099/mic.0.25645-0

[45] Iost I, Dreyfus M (1995) The stability of Escherichia coli lacZ mRNA depends upon the simultaneity of its synthesis and translation. EMBO J 14: 3252–3261. 7542588 10.1002/j.1460-2075.1995.tb07328.x PMC394387

[46] Chen X, Denison L, Levy M, Ellington AD (2009) Direct selection for ribozyme cleavage activity in cells. RNA 15: 2035–2045. 19776159 10.1261/rna.1635209 PMC2764470

[47] Ayre BG, Kohler U, Turgeon R, Haseloff J (2002) Optimization of trans-splicing ribozyme efficiency and specificity by in vivo genetic selection. Nucleic Acids Res 30: e141. 12490732 10.1093/nar/gnf141 PMC140090

[48] Guo F, Cech TR (2002) In vivo selection of better self-splicing introns in Escherichia coli: the role of the P1 extension helix of the Tetrahymena intron. RNA 8: 647–658. 12022231 10.1017/s1355838202029011 PMC1370285

[49] Unwalla HJ, Li H, Li SY, Abad D, Rossi JJ (2008) Use of a U16 snoRNA-containing ribozyme library to identify ribozyme targets in HIV-1. Mol Ther 16: 1113–1119. 18388915 10.1038/mt.2008.54 PMC2775071

[50] Wieland M, Hartig JS (2008) Improved aptazyme design and in vivo screening enable riboswitching in bacteria. Angew Chem Int Ed Engl 47: 2604–2607. 18270990 10.1002/anie.200703700

[51] Cech TR, Damberger SH, Gutell RR (1994) Representation of the secondary and tertiary structure of group I introns. Nat Struct Biol 1: 273–280. 7545072 10.1038/nsb0594-273

