# Supplementary figures and images for: In Vivo Evolution of a Catalytic RNA Couples Trans-Splicing to Translation
Source: PLoS One. 2014 Jan 23;9(1):e86473. doi: 10.1371/journal.pone.0086473 (PMC3900562; doi:10.1371/journal.pone.0086473)

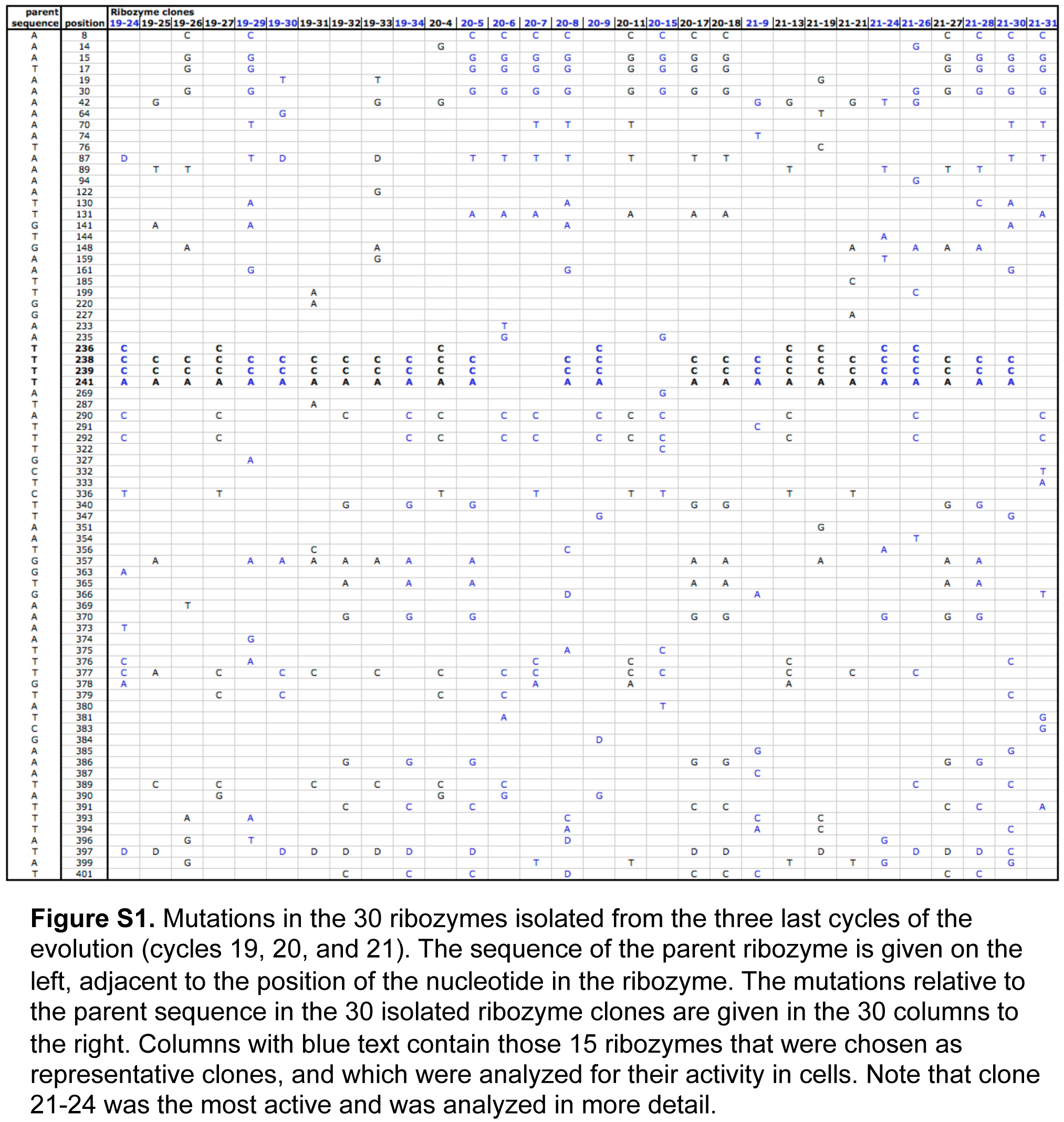

Supplement: Figure S1 — Mutations in the 30 ribozymes isolated from the three last cycles of the evolution (cycles 19, 20, and 21). (TIF) [file pone.0086473.s001.tif]

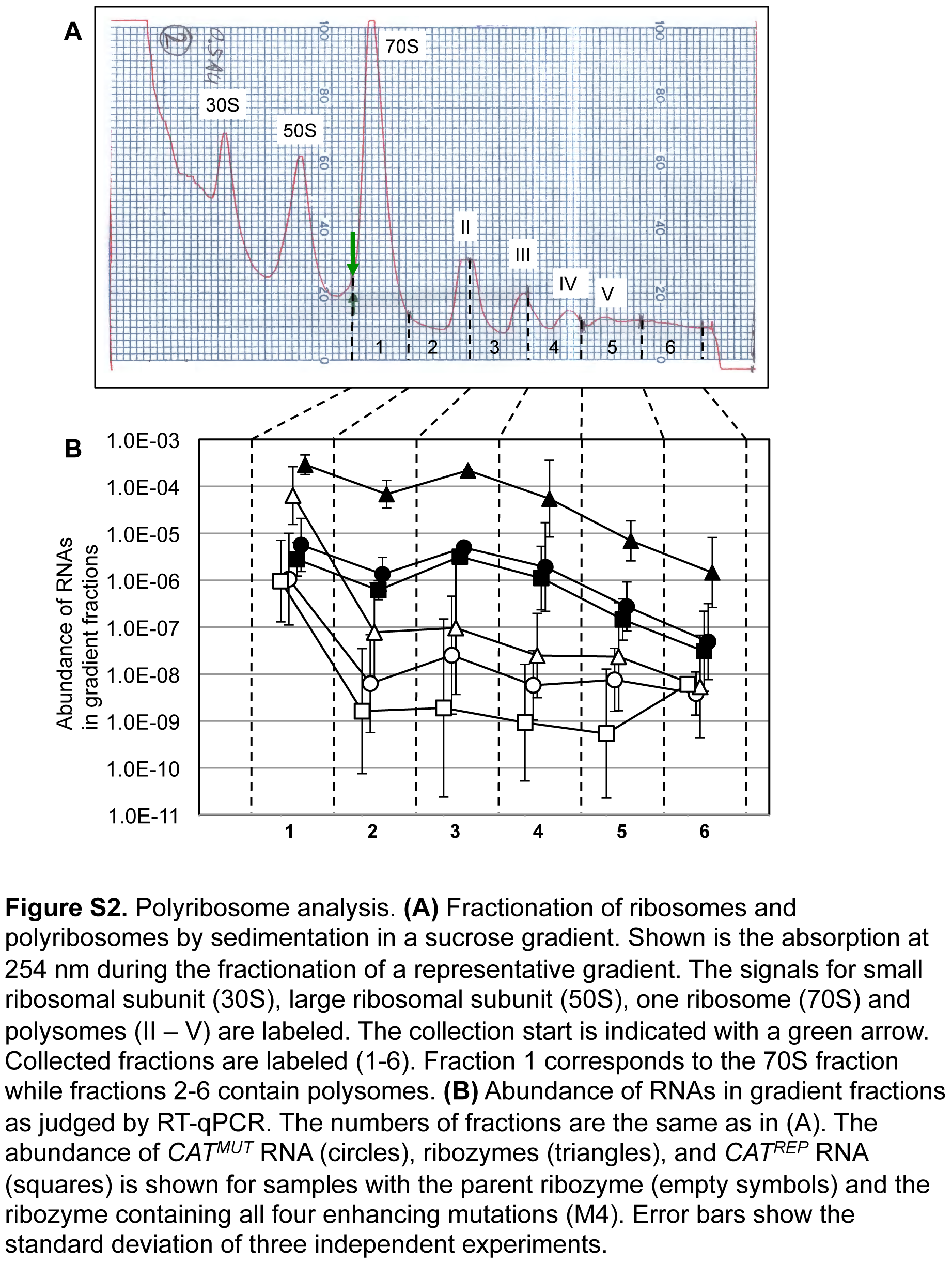

Supplement: Figure S2 — Polyribosome analysis. Shown is the absorption at 254 nm during the fractionation of ribosomes and polyribosomes on a representative sucrose gradient, and the abundance of RNAs in gradienmt fractions as judged by RT-qPCR. (TIF) [file pone.0086473.s002.tif]

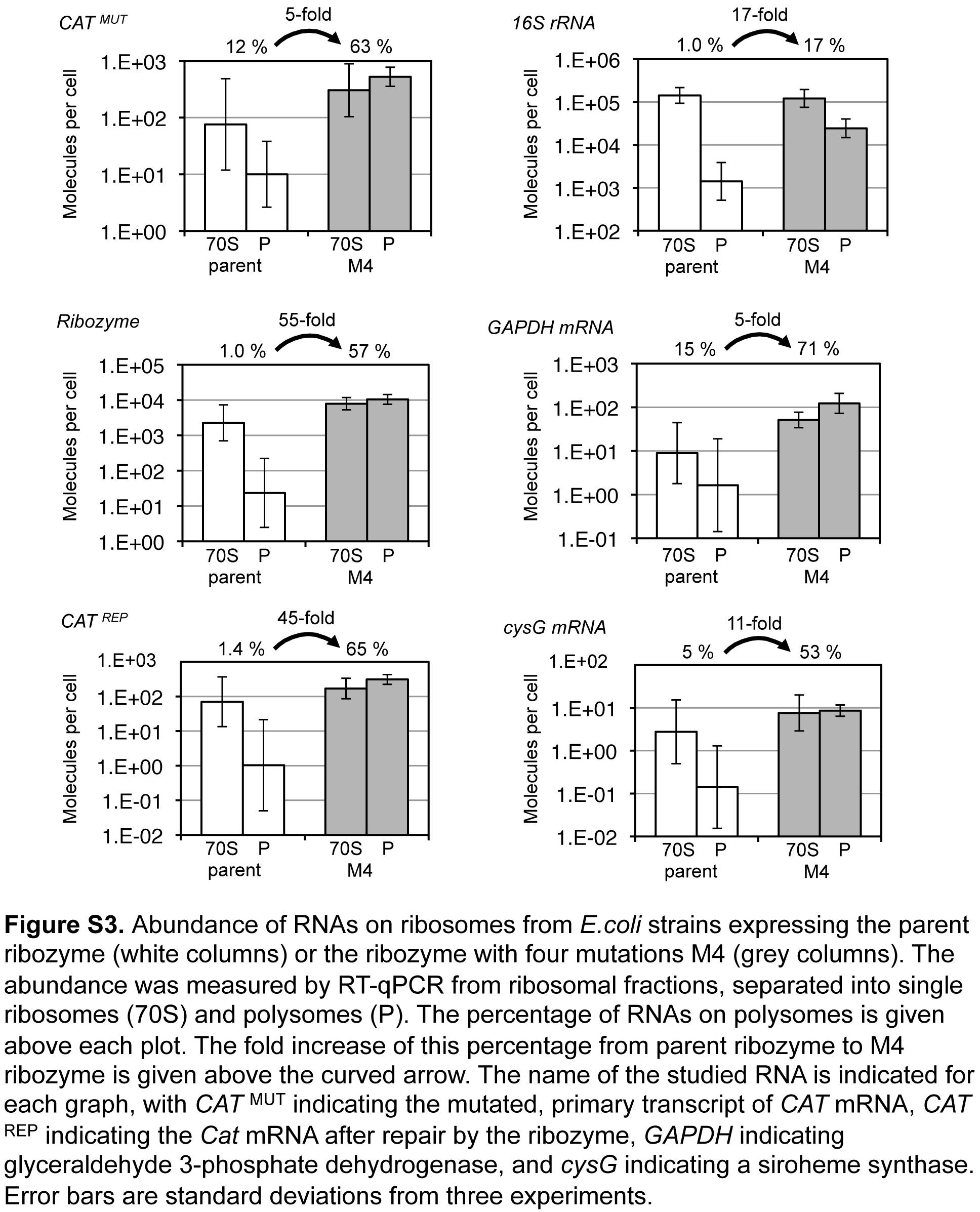

Supplement: Figure S3 — Abundance of RNAs on ribosomes from E. coli strains expressing the parent ribozyme or the M4 ribozyme. The percentage of RNAs on polysomes is given. Data are shown for the mutated, primary transcript of CAT mRNA, the ribozyme, the repaired CAT mRNA, 16S rRNA, and the mRNAs of GAPDH and cysG. (TIF) [file pone.0086473.s003.tif]
